# Association of School Social Status with COVID-19 Pandemic-Related Changes and Post-Pandemic Rebounds of Children’s Physical Fitness

**DOI:** 10.1186/s40798-025-00838-5

**Published:** 2025-04-23

**Authors:** Paula Teich, Fabian Arntz, Toni Wöhrl, Florian Bähr, Kathleen Golle, Reinhold Kliegl

**Affiliations:** 1 Faculty of Human Sciences, Research Focus Cognitive Sciences, University of Potsdam, Am Neuen Palais 10, 14469 Potsdam, Germany; 2 Faculty of Educational Sciences, Division of Sports and Movement Sciences, University of Erfurt, Erfurt, Germany

**Keywords:** Physical Fitness, Primary School Children, Regression Discontinuity Design, Linear Mixed Model, Socioeconomic Background

## Abstract

**Background:**

In a recent study, we examined Covid-19 pandemic effects on the physical fitness of German third-graders tested between 2016 and 2022. The present report includes new data from 2023 to examine whether there were post-pandemic rebounds in the negatively affected fitness components, and whether pandemic and potential rebound effects differed by school social status.

**Methods:**

The EMOTIKON project annually tests the fitness of all third-graders in the Federal State of Brandenburg, Germany. Tests assess cardiorespiratory endurance (6-min-run), coordination (star-run), speed (20-m linear sprint), lower (powerLOW, standing long jump), and upper (powerUP, ball-push test) limbs muscle power, and static balance (one-legged-stance test). A total of 108,308 third-graders aged between 8 and 9.2 years from 444 schools were tested in the falls from 2016 to 2023. Linear mixed models, specified for a regression discontinuity design with random factors for child and school, estimated pandemic effects on the first day of school in the school year 2020/21 (i.e., the critical date), as well as cohort trends before and after the pandemic onset.

**Results:**

Higher school social status was associated with better cardiorespiratory endurance, coordination, speed, and powerLOW. At the critical date, there were small negative pandemic effects in cardiorespiratory endurance, coordination, speed, and powerUP. Pandemic effects in speed and coordination were larger in schools with higher social status. Coordination and powerUP were characterized by a post-pandemic rebound, with slightly larger coordination rebounds for schools with higher social status. There was no evidence for rebounds of cardiorespiratory endurance and speed.

**Conclusions:**

Absence of evidence for task-specific rebounds may indicate long-term consequences of pandemic-related movement restrictions. Lower cardiorespiratory endurance, coordination, speed, and powerLOW in schools with low social status may indicate the need for improved access to sports opportunities in these schools.

## Key Points

• Pandemic effects were small and negative in cardiorespiratory endurance, coordination, speed, and upper limbs muscle power (powerUP); coordination and powerUP improved after the pandemic.

• Lower school social status was associated with poorer cardiorespiratory endurance, coordination, speed, and lower limbs muscle power (powerLOW). Pandemic effects in speed and coordination were larger in schools with higher social status; these schools also exhibited larger coordination rebounds than schools with lower social status.

• Results show the importance of environments and infrastructure providing sports opportunities for children’s fitness development; particularly children in low social status schools might benefit from improved access to sports opportunities.

## Background

Various studies have examined Covid-19-pandemic-related changes in children’s physical fitness [1–9]. Our recent study examined the physical fitness of third-graders tested between 2016 and 2022 and reported negative pandemic effects in three running tests. Here, we include new data from cohort 2023 to examine whether there were post-pandemic rebounds in the negatively affected fitness components, and whether pandemic and potential rebound effects differed by school social status.

Using the EMOTIKON test battery [10], 98,510 third-graders aged between 8 and 9 years were tested between 2016 and 2022 in the German Federal State of Brandenburg [8]. A regression discontinuity design (RDD) [11, 12], specified for a linear mixed model with random factors child and school, estimated pandemic effects on the first day of school in school year 2020/21 and found small negative pandemic effects in the 6-min run, star-run coordination test, and 20-m sprint [8]. Similar results were reported in Berlin, Germany, where 68,996 third-graders completed the German Motor Fitness Test [13] between school years 2011/12 and 2021/22. Children tested after the start of the pandemic exhibited lower performance in the 20-m sprint, side jumps, standing long jump and push-ups, relative to predicted performance taking into account pre-pandemic secular trends. However, children of the pandemic cohorts exhibited better 6-min run and sit-up performance [7]. In the Federal State of Thuringia, Germany, 38,084 third-graders were tested between school years 2017/18 and 2023/24 using the same fitness tests as in Brandenburg [1]. Pandemic effects on the first school day in school year 2020/21 were small and negative for cardiorespiratory endurance, coordination, lower, and upper limbs muscle power.

Some studies reported that Covid pandemic effects differed between schools and regions, and may have been moderated by indicators of schools’ social structure [7, 9, 14]. In general, children’s health and development are influenced by their living environments [15–18] including the family, school, and geographical region. For instance, socioeconomic status, often assessed with indicators like occupation, income or education levels [19], can influence children’s physical activity [18–20] and fitness [7, 9, 21]: Access to organized sports depends on financial resources and regional sports opportunities, and children are less likely to be in sports clubs if they come from families of lower socioeconomic status [20, 22]. Further, low socioeconomic status is related to limited access to healthy, more expensive food options, which may lead to adverse eating habits [23]. In high-income countries, low socioeconomic status is thus associated with a higher risk of childhood obesity [23, 24].

Some studies have used school-specific social indices, which include information like parental income/employment rates, migration background and special educational needs rates, to assess relationships between school social structure and children’s physical fitness [7, 9, 14]. In Berlin, Germany, children from schools with higher social disadvantage according to this index (i.e., lower social status), tended to exhibit higher BMIs [14] and poorer performance in tests assessing cardiorespiratory endurance, speed, coordination and muscular power/strength [7] compared to children from schools with higher social status. Pandemic-related increases in BMI were larger in schools of low social status in two German studies [9, 14], thus increasing social disparities. However, some studies of fitness test performance found the opposite pattern of results. In Berlin, children from schools with higher social status according to the school-specific social index exhibited larger pandemic-related declines in average fitness when adjusting for pre-pandemic secular trends [7]. Similar findings were reported in the German Federal States of Brandenburg and Thuringia, where higher average school fitness was associated with more pronounced pandemic effects [1, 8]. In contrast to these results, Wessely et al. [9] reported larger decreases in 6-min run performance for schools with lower social status.

In the Federal State of Brandenburg, pandemic-related changes in children’s physical fitness have not yet been analyzed with reference to schools’ social structure. Further, while there are many studies on pandemic-related changes in children’s fitness, it is still unclear how children’s fitness has developed after the pandemic and whether potential rebound effects differ by social status. Until 2022, there was little evidence for post-pandemic rebounds of cardiorespiratory endurance and speed in the Federal State of Brandenburg [8]. A reason for the absence of rebound effects may be that third-graders in cohorts 2020, 2021, and 2022 all experienced pandemic-related lockdowns and school closures in third, second, or first grade, respectively, and were not able to compensate for this loss of structured exercise. However, cohort 2023 was the first cohort that did not experience strict pandemic-related lockdowns in their school time (i.e., third-graders of cohort 2023 were enrolled to school in fall of 2021), and this cohort may thus exhibit better physical fitness relative to the previous years. The goal of the present paper was thus to examine whether (1) physical fitness and (2) pandemic effects differed by schools’ social structure, (3) whether negatively affected physical fitness components have recovered after the pandemic, and (4) whether potential rebound effects differed by school social structure.

Based on previous studies reporting lower physical fitness for children in schools or regions of lower socioeconomic background [7, 9, 25], we expected lower cardiorespiratory endurance, coordination, speed, and lower limbs muscle power in schools with lower social status.

As prior studies indicated more pronounced pandemic effects for “fitter” schools [1, 7, 8], we expected larger negative pandemic effects for schools with a higher social status. After the pandemic, movement restrictions were lifted and access to school sports and sports clubs was restored. If there is evidence for post-pandemic rebound effects, potential rebounds may thus have been larger in schools with higher social status, located in more affluent areas where children likely have easier access to organized sports clubs.

Secondary analyses tested whether previously reported effects of age [26] on children’s physical fitness are moderated by schools’ social status. Previous research has shown that third-graders in “fitter” schools exhibit larger age-related fitness trends within the ninth year of life [26]. Fühner et al. suggested that fitter schools may be located in more affluent areas and be attended by active children with greater access to sports opportunities promoting larger age gains. Further, they may conduct more effective physical education classes and extracurricular school sports that drive larger age-related development [26]. If higher school social status is associated with better average physical fitness, higher social status may also be associated with larger age-related fitness development in the ninth year of life.

## Methods

### Experimental approach

In the Federal State of Brandenburg, Germany, the EMOTIKON project annually tests the physical fitness of all third graders (www.uni-potsdam.de/en/emotikon/). The project is mandated and approved by the Ministry of Education, Youth and Sport of the Federal State of Brandenburg, Germany and is obligatory for all public primary schools [27]. The data collection period is between August and December; physical fitness tests are conducted by physical education teachers. Before test administration, parents receive written information on the physical fitness tests to be conducted and on data processing regulations; written parental consent is not required due to the obligatory nature of the assessments. Researchers received the data completely anonymized from the Ministry of Education, Youth and Sport of the Federal State of Brandenburg, Germany. No personally identifiable information on the children was available to the researchers at any point.

### Population

We used data from cohorts 2016 to 2022 that were analyzed and published previously [8, 26], and added new data from children who were tested in the fall of 2023. Analyses focused on third-graders who had been enrolled to school according to the legal key date and were aged between 8.0 and 9.2 years. Analyses for third-graders with delayed school enrollment (older-than-key-age children, OTK) are reported in the Supplementary Material in the OSF repository (https://osf.io/5273n/) [28]. The two groups were analyzed separately because cross-sectional physical fitness development in third grade is qualitatively different for key-age and OTK children [26, 29].

The *school-specific social index* was provided by the Ministry of Education, Youth and Sport of the Federal State of Brandenburg, Germany, with the goal of distributing school budget according to schools’ socio-structural disadvantage. The school social index was computed based on indicators of social marginalization, namely the (1) social welfare rate (German: SGB-II) weighted by student residential commune (for independent cities: district rates), (2) proportion of students with non-German primary language, and (3) proportion of students with special educational needs [30, 31]. We refer to schools as having “high social status” when they fall into the category of low social disadvantage (and vice versa) to maintain the traditional polarity with socioeconomic status and improve clarity of the text. Based on quartiles of the weighted composite score computed from the three indicators, schools were categorized into four groups of “school social status”. Category 1 included schools with the highest social status, and category 4 included schools with the lowest social status. Schools’ social status categories are freely available online [30, 31]. For analysis, we used the four-level school social status factor.

### Physical fitness tests

Details on test administration are reported in previous studies [1, 8, 26]. Tests assessed the six physical fitness components (also referred to as physical fitness dimensions [13, 32]) cardiorespiratory endurance, coordination, speed, lower and upper limbs muscle power, and static balance. *Cardiorespiratory endurance* was tested by the 6-min run. The dependent variable was distance traveled in meters. *Coordination* was tested using the star-run test, in which children had to memorize and complete a star-shaped parkour as fast as possible using different movement forms (running forward/backward, sidesteps to the right/left). The dependent variable was time measured in seconds, which was transformed to meters/second for analysis. *Speed* was tested using the 20-m linear sprint. Again, the dependent variable was time measured in seconds, which was transformed to meters/second for analysis. *Lower limbs muscle power* (powerLOW) was tested by the standing long jump, with the dependent variable distance measured in centimeters. *Upper limbs muscle power* (powerUP) was assessed by the ball-push test, where children had to push a 1-kg medicine ball from a standing position as far as possible. Pushing distance was measured in meters to the nearest 10 centimeters. *Static balance* was tested using the one-legged stance test with eyes closed, with the dependent variable time measured in seconds (logarithmic transformation for analysis).

### Statistics

For data preprocessing, we used the tidyverse packages [33] in *R version 4.2.3* [34], and the *R Studio IDE* [35]. We fitted Linear Mixed Models (LMMs) using the *MixedModels.jl* [36] and *MixedModelsMakie.jl* [37] packages in *Julia* (version 1.10) [38].

Preprocessing of data was adapted from previous studies [26, 39]. According to a Box-Cox distributional analysis [40], transformation of test sores of the star-run and the 20-m sprint to meters/seconds (reciprocal transformation multiplied by running distance) brought model residuals in line with a normal distribution. Test scores of the one-legged stance test were log-transformed.

Data selection for data from cohorts 2016–2022 is documented in the previous report [8]. For data from 2023, we followed the same procedure. We first computed z-scores separately for each test and for boys and girls and excluded test scores outside of a ± 3 SD range. For the one-legged stance test assessing static balance, we did not exclude scores outside of the ± 3 SD range, since for this test, the whole range of possible test scores with a maximum of 60 s indicated valid performance [8, 39]. In a second step, we recomputed z-scores for all data using test means and SDs of keyage children analyzed in the previous report (i.e., cohorts 2016–2022) [8]. This was done to obtain the same z-scores for data from cohorts 2016–2022 as in the previously published analysis [8]. We only kept data from schools with available social index (31,239 test scores from 5494 children in 75 schools were excluded). We ended up with 628,399 test scores from 108,308 children in 444 schools. Table 1 provides an overview of the sample characteristics. A more detailed sample description including test means and SDs in original metric is provided in the OSF repository [28].

**Table 1:** Sample description

Cohorts	*N* children (% girls)	*N* test scores	Age [years] M (SD)	*N* schools
2016–2019	51,796 (51%)	300,778	8.61 (0.28)	435
2020–2022	41,798 (51%)	242,073	8.56 (0.28)	436
2023	14,714 (52%)	85,548	8.61 (0.28)	432
*Total*	108,308 (51%)	628,399	8.59 (0.28)	444

Physical fitness tests were treated as six levels of the factor “physical fitness component” (i.e., cardiorespiratory endurance, coordination, speed, powerLOW, powerUP, and static balance). For the school-specific four-level factor “social status” (category 1 indicates highest, and category 4 lowest social status) we specified a Helmert contrast comparing (1) the mean of categories 1, 2, and 3 against category 4 (SI123 vs. SI4), (2), the mean of categories 1 and 2 against category 3 (SI12 vs. SI3), and (3) category 1 against category 2 (SI1 vs. SI2).

The Covid factor was dummy coded, with the pre-pandemic cohorts (i.e., 2016–2019) as the reference category coded as “0”, and cohorts 2020–2023 coded as “1”. For the factor Gender, we specified a sequential difference contrast with positive estimates indicating better performance of boys. A regression discontinuity design [11, 12] tested Covid pandemic effects at the first day of school in school year 2020/21 (i.e., August 10 in the Federal State of Brandenburg, Germany) and secular trends (linear, quadratic) before and after this critical date. Test date was centered at the critical date, and age was centered at 8.5 years.

The RDD was specified for a LMM with random factors child (*N* = 108,308) and school (*N* = 444). Details on parsimonious model selection are reported in script *keyage_lmm_16_23.qmd* in the OSF repository. We started with a complex LMM with fixed effects of Covid, school social index, age (linear), gender, and linear and quadratic test date trends (i.e., cohort trends, nested under the Covid factor), with all terms nested under the six levels of ‘physical fitness component’. In this model, social status interacted with Covid, linear and quadratic test date trends, and age. For the random factor child, we included variance components (VCs) and correlation parameters (CPs) of physical fitness component, and for the random factor school, we included VCs and CPs of physical fitness component, Covid, test date trends (linear, quadratic), age, and gender effects. To obtain a parsimonious LMM that was supported by the data without overparameterization [41], we reduced the complexity of the fixed and random effect structure (details reported in the OSF repository). The final LMM included the fixed effects reported above, with school social index interacting with Covid, linear test date trends before and after Covid onset, quadratic test date trends after the pandemic onset, and with age. For the random factor child, we included VCs and CPs of physical fitness component. For the random factor school, the LMM included VCs of physical fitness component, Covid nested under the levels of physical fitness component, gender and CPs for all VCs except for gender. Following earlier practice [1, 8, 29], |z-values| ≥ 2.0 were interpreted as statistically significant.

## Results

**Fig. 1:**
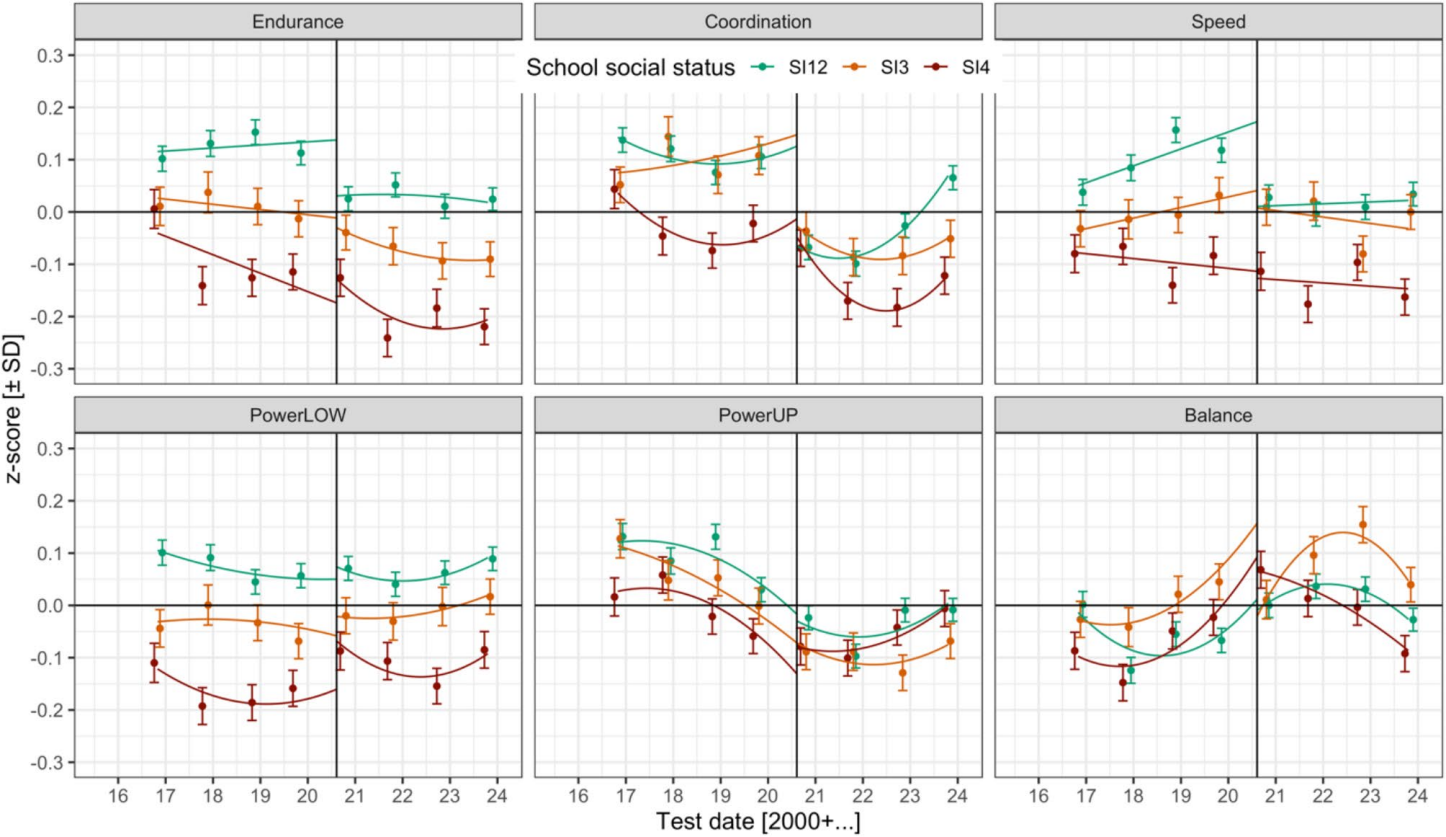
Physical fitness by fitness component, school social status, and cohort (means and 95% CIs). The vertical line marks the critical date (first day of school in school year 2020/21). Endurance (i.e., cardiorespiratory endurance) = 6-min run, Coordination = star-run, Speed = 20-m linear sprint, PowerLOW (i.e., lower limbs muscle power) = standing long jump, PowerUP (i.e., upper limbs muscle power) = ball-push test, Balance = one-legged-stance with eyes closed. SI12 = school social index categories 1 and 2 (high social status), SI3 = school social index category 3, SI4 = school social index category 4 (lowest social status). For visual clarity, school social index categories 1 and 2 were combined. A figure depicting physical fitness for all four social index categories is provided in the Supplementary Material in the OSF repository

Figure 1 shows mean test performance by physical fitness component, cohort (2016–2023), and school social index category. In this figure, school social index categories 1 and 2 were combined to enhance visual clarity. LMM-based inferential fixed effect estimates with standard errors and z-values are reported in Table 2.1 through 2.2, 2.3, 2.4, 2.5, 2.6 and Table 3; VCs and CPs related to the random factors child and school are presented in Table 4. While they are reported in different tables, effects were estimated in the same LMM including 628,399 observations from 108,308 children in 444 schools. The LMM estimated test date trends nested under the Covid factor (i.e., separate linear and quadratic cohort trends before and after the critical date). An overview of the LMM’s fixed effects, as well as tables with test scores by school social status and cohort in the original test metrics are provided in the Supplementary Material in the OSF repository [28].

*Main effects of school social status on physical fitness*. In line with our hypothesis, and as depicted in Fig. 1 and reported in Table 2, children in schools with higher social status exhibited better performances in the 6-min run (cardiorespiratory endurance, SI123 vs. SI4: b = 0.166, z = 3.69; SI12 vs. SI3: b = 0.145, z = 3.03), star-run (coordination, SI123 vs. SI4: b = 0.114, z = 2.28), 20 m-sprint (speed, SI123 vs. SI4: b = 0.196, z = 4.37; SI1 vs. SI2: b = 0.114, z = 2.07), and standing long jump (powerLOW, SI123 vs. SI4: b = 0.143, z = 3.93; SI12 vs. SI3: b = 0.095, z = 2.48; SI1 vs. SI2: b = 0.179, z = 4.03) compared to schools with lower social status. An additional result was that schools of social status category 3 exhibited better average one-legged-stance performance (static balance) than schools with social status categories 1 and 2 (SI12 vs. SI3: b = -0.132, z = -2.80).

### Covid Pandemic Effects and Cohort Trends of Physical Fitness

*Cardiorespiratory endurance* (6-min run). When tested at the first day of school in school year 2020/21 indicated by the vertical line in Fig. 1, there was a small negative Covid pandemic effect (b = -0.054, z = -2.05). As shown in Table 2.1, there was no evidence that pandemic effects at the critical date differed between schools of different social index categories (|z| < 2). Although these interactions were not significant, Fig. 1 suggests that pandemic effects were larger for school status categories 1 and 2 (high social status) compared to categories 3 and 4 (lower social status). Indeed, in a post-hoc LMM without Covid-VCs and CPs in the random effect structure for schools, the fixed effect interactions between school social status and Covid pandemic effect became significant also for cardiorespiratory endurance (SI123 vs. SI4: b = -0.081, z = -2.66; SI12 vs. SI3: b = -0.085, z = -2.62), with schools of higher social status exhibiting larger negative pandemic effects than schools with lower social status (code provided in OSF repository [28]).

Between 2016 and 2019, main effects of linear and quadratic cohort trends were not significant. After the start of the Covid-19 pandemic, 6-min run performance declined (b = -0.043, z = -3.24), followed by a plateau (b = 0.009, z = 2.45). Cohort trends differed by school social status. Schools with higher social status exhibited slightly more positive pre-pandemic linear secular trends than schools with lower social status (SI123 vs. SI4: b = 0.029, z = 3.31; SI12 vs. SI3: b = 0.020, z = 2.14; SI1 vs. SI2: b = 0.032, z = 3.08). After the pandemic, schools with social status category 1 (highest social status) exhibited a less negative linear cohort trend (SI1 vs. SI2: b = 0.129, z = 3.51) and a more negative quadratic cohort trend (SI1 vs. SI2: b = -0.042, z = -3.99), compared to schools of status category 2.

*Coordination* (star-run). As shown in Fig. 1 and Table 2.2, there was a small negative pandemic effect at the critical date (b = -0.189, z = -6.28). This pandemic effect was more pronounced in schools with higher social status compared to schools with low social status (SI123 vs. SI4: b = -0.145, z = -2.62). Before the pandemic onset, coordination was characterized by a positive linear (b = 0.106, z = 5.04) and quadratic (b = 0.024, z = 5.38) cohort trend. After the pandemic onset, star-run performance declined linearly (b = -0.062, z = -4.61), followed by a positive quadratic trend (b = 0.024, z = 6.21). Schools with high social status exhibited a more positive linear cohort trend after the pandemic onset compared to schools with low social status (SI123 vs. S4: b = 0.067, z = 2.13).

**Table 2.1:** Cardiorespiratory endurance (6-min run). Fixed effect estimates, standard errors (SE), and z-values of the linear mixed model for Covid pandemic effects, cohort trends, school social status and their interactions

	Estimate (b)	SE	z-value
Endurance Intercept	0.017	0.027	0.65
*School social status*			
SI123 vs. SI4	0.166	0.045	**3.69**
SI12 vs. SI3	0.145	0.048	**3.03**
SI1 vs. SI2	0.055	0.055	1.00
*Test date / Covid effects and interactions with school social status*			
*Pre-covid td1*	0.022	0.021	1.08
Pre-covid td1 x SI123 vs. SI4	0.029	0.009	**3.31**
Pre-covid td1 x SI12 vs. SI3	0.020	0.009	**2.14**
Pre-covid td1 x SI1 vs. SI2	0.032	0.010	**3.08**
*Pre-covid td2*	0.005	0.004	1.21
*Covid @ critical date*	-0.054	0.026	**-2.05**
Covid x SI123 vs. SI4	-0.061	0.045	-1.36
Covid x SI12 vs. SI3	-0.088	0.048	-1.85
Covid x SI1 vs. SI2	-0.057	0.055	-1.05
*Post-covid td1*	-0.043	0.013	**-3.24**
Post-covid td1 x SI123 vs. SI4	0.045	0.031	1.45
Post-covid td1 x SI12 vs. SI3	0.022	0.033	0.68
Post-covid td1 x SI1 vs. SI2	0.129	0.037	**3.51**
*Post-covid td2*	0.009	0.004	**2.45**
Post-covid td2 x SI123 vs. SI4	-0.009	0.009	-1.04
Post-covid td2 x SI12 vs. SI3	-0.004	0.009	-0.37
Post-covid td2 x SI1 vs. SI2	-0.042	0.011	**-3.99**

Note. School social index contrasts: SI123 vs. SI4 = Mean of index categories 1, 2, and 3 vs. index category 4; SI12 vs. SI3 = Mean of index categories 1 and 2 vs. index category 3, SI1 vs. SI2 = Index category 1 vs. index category 2. Covid @ critical date = Covid pandemic effect estimated at first school day in school year 2020/21. Pre-covid = before critical date, post-covid = after critical date. Td1 = linear test date trend, td2 = quadratic test date trend. Bold = |z| > 2

**Table 2.2:** Coordination (star-run). Fixed effect estimates, standard errors (SE), and z-values of the linear mixed model for Covid pandemic effects, cohort trends, school social status and their interactions

	Estimate (b)	SE	z-value
Coordination Intercept	0.119	0.028	**4.19**
*School social status*			
SI123 vs. SI4	0.114	0.050	**2.28**
SI12 vs. SI3	-0.011	0.053	-0.20
SI1 vs. SI2	0.062	0.062	1.01
*Test date / Covid effects and interactions with school social status*			
*Pre-covid td1*	0.106	0.021	**5.04**
Pre-covid td1 x SI123 vs. SI4	0.005	0.009	0.56
Pre-covid td1 x SI12 vs. SI3	-0.007	0.009	-0.73
Pre-covid td1 x SI1 vs. SI2	0.018	0.011	1.75
*Pre-covid td2*	0.024	0.004	**5.38**
*Covid @ critical date*	-0.189	0.030	**-6.28**
Covid x SI123 vs. SI4	-0.145	0.055	**-2.62**
Covid x SI12 vs. SI3	-0.054	0.059	-0.92
Covid x SI1 vs. SI2	-0.034	0.068	-0.50
*Post-covid td1*	-0.062	0.013	**-4.61**
Post-covid td1 x SI123 vs. SI4	0.067	0.032	**2.13**
Post-covid td1 x SI12 vs. SI3	0.037	0.034	1.10
Post-covid td1 x SI1 vs. SI2	0.003	0.037	0.09
*Post-covid td2*	0.024	0.004	**6.21**
Post-covid td2 x SI123 vs. SI4	-0.005	0.009	-0.60
Post-covid td2 x SI12 vs. SI3	0.007	0.010	0.70
Post-covid td2 x SI1 vs. SI2	0.001	0.011	0.12

Note. School social index contrasts: SI123 vs. SI4 = Mean of index categories 1, 2, and 3 vs. index category 4; SI12 vs. SI3 = Mean of index categories 1 and 2 vs. index category 3, SI1 vs. SI2 = Index category 1 vs. index category 2. Covid @ critical date = Covid pandemic effect estimated at first school day in school year 2020/21. Pre-covid = before critical date, post-covid = after critical date. Td1 = linear test date trend, td2 = quadratic test date trend. Bold = |z| > 2

*Speed* (20 m sprint). When estimated on the first day of school in school year 2020/21 (indicated by the vertical line in Fig. 1), 20 m sprint performance was characterized by a small negative Covid pandemic effect (b = -0.087, z = -3.08). This pandemic effect was more pronounced for schools of higher social status (SI12 vs. SI3: b = -0.140, z = -2.65; SI1 vs. SI2: b = -0.175, z = -2.88). Before the pandemic, sprint performance slightly increased (linear cohort trend, b = 0.042, z = 2.01), with schools of higher social status exhibiting more positive linear cohort trends (SI123 vs. SI4: b = 0.031, z = 3.55; SI1 vs. SI2: b = 0.042, z = 3.96). After the pandemic onset, there was no evidence for significant linear or quadratic cohort trends (|z| < 2).

**Table 2.3:** Speed (20 m sprint). Fixed effect estimates, standard errors (SE), and z-values of the linear mixed model for Covid pandemic effects, cohort trends, school social status and their interactions

	Estimate (b)	SE	z-value
Speed Intercept	0.032	0.027	1.20
*School social status*			
SI123 vs. SI4	0.196	0.045	**4.37**
SI12 vs. SI3	0.095	0.048	1.98
SI1 vs. SI2	0.114	0.055	**2.07**
*Test date / Covid effects and interactions with school social status*			
Pre-covid td1	0.042	0.021	**2.01**
Pre-covid td1 x SI123 vs. SI4	0.031	0.009	**3.55**
Pre-covid td1 x SI12 vs. SI3	0.015	0.009	1.64
Pre-covid td1 x SI1 vs. SI2	0.042	0.011	**3.96**
Pre-covid td2	0.005	0.004	1.03
Covid @ critical date	-0.087	0.028	**-3.08**
Covid x SI123 vs. SI4	-0.081	0.050	-1.63
Covid x SI12 vs. SI3	-0.140	0.053	**-2.65**
Covid x SI1 vs. SI2	-0.175	0.061	**-2.88**
Post-covid td1	-0.010	0.013	-0.71
Post-covid td1 x SI123 vs. SI4	-0.016	0.031	-0.50
Post-covid td1 x SI12 vs. SI3	0.051	0.033	1.53
Post-covid td1 x SI1 vs. SI2	0.003	0.037	0.09
Post-covid td2	0.002	0.004	0.58
Post-covid td2 x SI123 vs. SI4	0.009	0.009	1.01
Post-covid td2 x SI12 vs. SI3	-0.010	0.010	-1.04
Post-covid td2 x SI1 vs. SI2	0.005	0.011	0.45

Note. School social index contrasts: SI123 vs. SI4 = Mean of index categories 1, 2, and 3 vs. index category 4; SI12 vs. SI3 = Mean of index categories 1 and 2 vs. index category 3, SI1 vs. SI2 = Index category 1 vs. index category 2. Covid @ critical date = Covid pandemic effect estimated at first school day in school year 2020/21. Pre-covid = before critical date, post-covid = after critical date. Td1 = linear test date trend, td2 = quadratic test date trend. Bold = |z| > 2

*PowerLOW* (standing long jump). When estimated at the critical date, there was no evidence for a Covid-19 pandemic effect on standing long jump performance (|z| < 2). Between 2016 and 2019, standing long jump performance was characterized by a positive linear (b = 0.058, z = 2.71) and quadratic (b = 0.016, z = 3.40) cohort trend. The positive linear cohort trend was more pronounced for schools with social status category 1 (highest social status) compared to schools in category 2 (b = 0.053, z = 4.90). After the pandemic onset, standing long jump performance decreased linearly (b = -0.050, z = -3.66), followed by a positive quadratic trend (b = 0.016, z = 3.96). There was no evidence that these cohort trends differed significantly between school social status categories.

**Table 2.4:** PowerLOW (standing long jump). Fixed effect estimates, standard errors (SE), and z-values of the linear mixed model for Covid pandemic effects, cohort trends, school social status and their interactions

	Estimate (b)	SE	z-value
PowerLOW Intercept	-0.026	0.024	-1.07
*School social status*			
SI123 vs. SI4	0.143	0.036	**3.93**
SI12 vs. SI3	0.095	0.038	**2.48**
SI1 vs. SI2	0.179	0.044	**4.03**
*Test date / Covid effects and interactions with school social status*			
Pre-covid td1	0.058	0.022	**2.71**
Pre-covid td1 x SI123 vs. SI4	0.000	0.009	-0.04
Pre-covid td1 x SI12 vs. SI3	-0.006	0.010	-0.66
Pre-covid td1 x SI1 vs. SI2	0.053	0.011	**4.90**
Pre-covid td2	0.016	0.005	**3.40**
Covid @ critical date	0.020	0.026	0.80
Covid x SI123 vs. SI4	-0.025	0.041	-0.62
Covid x SI12 vs. SI3	-0.003	0.043	-0.07
Covid x SI1 vs. SI2	-0.079	0.049	-1.59
Post-covid td1	-0.050	0.014	**-3.66**
Post-covid td1 x SI123 vs. SI4	0.018	0.032	0.57
Post-covid td1 x SI12 vs. SI3	-0.033	0.034	-0.95
Post-covid td1 x SI1 vs. SI2	-0.020	0.038	-0.52
Post-covid td2	0.016	0.004	**3.96**
Post-covid td2 x SI123 vs. SI4	-0.003	0.009	-0.29
Post-covid td2 x SI12 vs. SI3	0.007	0.010	0.70
Post-covid td2 x SI1 vs. SI2	-0.007	0.011	-0.66

Note. School social index contrasts: SI123 vs. SI4 = Mean of index categories 1, 2, and 3 vs. index category 4; SI12 vs. SI3 = Mean of index categories 1 and 2 vs. index category 3, SI1 vs. SI2 = Index category 1 vs. index category 2. Covid @ critical date = Covid pandemic effect estimated at first school day in school year 2020/21. Pre-covid = before critical date, post-covid = after critical date. Td1 = linear test date trend, td2 = quadratic test date trend. Bold = |z| > 2

*PowerUP* (ball-push test). Ball-push test performance was characterized by a small negative Covid pandemic effect when estimated at the first day of school in school year 2020/21 (b = -0.066, b = -2.60). There was no evidence for significant differences in this Covid pandemic effect between schools of different social index categories. Between 2016 and 2019, ball-push test performance exhibited a small positive quadratic cohort trend (b = 0.010, z = 2.31). Between 2020 and 2023, ball-push test performance decreased linearly (b = -0.029, z = -2.24), followed by a rebound (i.e., positive quadratic trend, b = 0.010, z = 2.62; depicted in Fig. 1). Cohort trends largely did not differ between school social status categories. The only significant interaction between social status and cohort trend indicated that schools of social status category 1 exhibited a more positive linear cohort trend between 2020 and 2023 than schools of index category 2 (b = 0.031, z = 3.09).

*Balance* (one-legged-stance test). There was no evidence for a significant main effect of the Covid pandemic on one-legged-stance test performance at the critical date (|z| < 2). However, schools with social index categories 1 and 2 exhibited a more positive pandemic effect than schools of index category 3 (b = 0.187, z = 3.31). Prior to the pandemic, one-legged-stance performance was characterized by a positive linear (b = 0.101, z = 4.75) and quadratic (b = 0.021, z = 4.68) cohort trend. Schools with lower social status exhibited more pronounced linear trends (SI123 vs. SI4: b = -0.035, z = -3.93; SI12 vs. SI3: b = -0.039, z = -4.20, see Fig. 1). After the pandemic onset, performance initially increased linearly (0.066, z = 4.85), followed by a negative quadratic decline (b = -0.025, z = -6.39). During this period, the positive linear cohort trend was more pronounced in schools of status categories 1, 2, and 3, relative to category 4 (SI123 vs. SI4: b = 0.094, z = 2.95). Further, schools in category 1 exhibited a slightly more pronounced secular balance trend between 2020 and 2023 compared to schools in index category 2, indicated by a larger positive linear cohort trend (SI1 vs. SI2: b = 0.089, z = 2.39) and more pronounced (i.e., more negative) quadratic decline (SI1 vs. SI2: b = -0.026, z = -2.39). Similarly, schools in index category 3 exhibited a more pronounced secular trend compared to schools in index categories 1 and 2, indicated by a larger positive linear cohort trend (SI12 vs. SI3: b = -0.093, z = -2.75) and stronger quadratic decline (SI12 vs. SI3: b = 0.021, z = 2.20).

**Table 2.5:** PowerUP (Ball-push test). Fixed effect estimates, standard errors (SE), and z-values of the linear mixed model for Covid pandemic effects, cohort trends, school social status and their interactions

	Estimate (b)	SE	z-value
PowerUP Intercept	0.000	0.024	0.00
*School social status*			
SI123 vs. SI4	0.055	0.036	1.52
SI12 vs. SI3	0.070	0.038	1.82
SI1 vs. SI2	0.045	0.044	1.02
*Test date / Covid effects and interactions with school social status*			
Pre-covid td1	0.023	0.020	1.12
Pre-covid td1 x SI123 vs. SI4	-0.006	0.009	-0.66
Pre-covid td1 x SI12 vs. SI3	0.017	0.009	1.88
Pre-covid td1 x SI1 vs. SI2	0.031	0.010	**3.09**
Pre-covid td2	0.010	0.004	**2.31**
Covid @ critical date	-0.066	0.025	**-2.60**
Covid x SI123 vs. SI4	-0.032	0.043	-0.76
Covid x SI12 vs. SI3	-0.044	0.045	-0.98
Covid x SI1 vs. SI2	-0.031	0.052	-0.59
Post-covid td1	-0.029	0.013	**-2.24**
Post-covid td1 x SI123 vs. SI4	-0.022	0.030	-0.73
Post-covid td1 x SI12 vs. SI3	0.014	0.032	0.43
Post-covid td1 x SI1 vs. SI2	-0.043	0.036	-1.21
Post-covid td2	0.010	0.004	**2.62**
Post-covid td2 x SI123 vs. SI4	0.002	0.009	0.24
Post-covid td2 x SI12 vs. SI3	0.000	0.009	0.05
Post-covid td2 x SI1 vs. SI2	0.008	0.010	0.78

Note. School social index contrasts: SI123 vs. SI4 = Mean of index categories 1, 2, and 3 vs. index category 4; SI12 vs. SI3 = Mean of index categories 1 and 2 vs. index category 3, SI1 vs. SI2 = Index category 1 vs. index category 2. Covid @ critical date = Covid pandemic effect estimated at first school day in school year 2020/21. Pre-covid = before critical date, post-covid = after critical date. Td1 = linear test date trend, td2 = quadratic test date trend. Bold = |z| > 2

**Table 2.6:** Static balance (one-legged-stance with eyes closed). Fixed effect estimates, standard errors (SE), and z-values of the linear mixed model for Covid pandemic effects, cohort trends, school social status and their interactions

	Estimate (b)	SE	z-value
Balance Intercept	0.050	0.027	1.89
*School social status*			
SI123 vs. SI4	-0.020	0.045	-0.46
SI12 vs. SI3	-0.132	0.047	**-2.80**
SI1 vs. SI2	0.017	0.055	0.31
*Test date / Covid effects and interactions with school social status*			
Pre-covid td1	0.101	0.021	**4.75**
Pre-covid td1 x SI123 vs. SI4	-0.035	0.009	**-3.93**
Pre-covid td1 x SI12 vs. SI3	-0.039	0.009	**-4.20**
Pre-covid td1 x SI1 vs. SI2	0.002	0.011	0.15
Pre-covid td2	0.021	0.005	**4.68**
Covid @ critical date	-0.047	0.029	-1.60
Covid x SI123 vs. SI4	-0.055	0.053	-1.03
Covid x SI12 vs. SI3	0.187	0.057	**3.31**
Covid x SI1 vs. SI2	0.053	0.065	0.82
Post-covid td1	0.066	0.014	**4.85**
Post-covid td1 x SI123 vs. SI4	0.094	0.032	**2.95**
Post-covid td1 x SI12 vs. SI3	-0.093	0.034	**-2.75**
Post-covid td1 x SI1 vs. SI2	0.089	0.037	**2.39**
Post-covid td2	-0.025	0.004	**-6.39**
Post-covid td2 x SI123 vs. SI4	-0.015	0.009	-1.64
Post-covid td2 x SI12 vs. SI3	0.021	0.010	**2.20**
Post-covid td2 x SI1 vs. SI2	-0.026	0.011	**-2.39**

Note. School social index contrasts: SI123 vs. SI4 = Mean of index categories 1, 2, and 3 vs. index category 4; SI12 vs. SI3 = Mean of index categories 1 and 2 vs. index category 3, SI1 vs. SI2 = Index category 1 vs. index category 2. Covid @ critical date = Covid pandemic effect estimated at first school day in school year 2020/21. Pre-covid = before critical date, post-covid = after critical date. Td1 = linear test date trend, td2 = quadratic test date trend. Bold = |z| > 2

*Age- and gender-related differences in physical fitness*. Previously reported age and gender effects [1, 8, 26, 39] were replicated and are shown in Table 3. As hypothesized, age-related gains in five of six physical fitness tests were larger in schools with higher social status (social index categories 1–3) than in schools with low social status (index category 4). This was the case for performance in the 6-min run (b = 0.101, z = 4.24), star-run (b = 0.072, z = 2.98), 20 m sprint (b = 0.091, z = 3.78), standing long jump (b = 0.096, z = 3.90), and ball-push test (b = 0.066, z = 2.87). Further, schools with social index categories 1 and 2 exhibited larger age effects in the ball-push test than schools with index category 3 (b = 0.065, z = 2.64). Figures depicting these differences in age-related development are provided in the Supplementary Material in the OSF repository [28].

**Table 3:** Fixed effect estimates, standard errors (SE), and z-values of the linear mixed model for age and gender effects, as well as age by school social status interactions

	Estimate (b)	SE	z-value
*Cardiorespiratory endurance (6-min run)*			
Age	0.081	0.010	**7.99**
Age x SI123 vs. SI4	0.101	0.024	**4.24**
Age x SI12 vs. SI3	-0.001	0.025	-0.05
Age x SI1 vs. SI2	0.040	0.028	1.43
Gender	0.443	0.006	**74.14**
*Coordination (star-run)*			
Age	0.278	0.010	**26.94**
Age x SI123 vs. SI4	0.072	0.024	**2.98**
Age x SI12 vs. SI3	0.040	0.025	1.57
Age x SI1 vs. SI2	0.000	0.028	0.02
Gender	0.230	0.006	**38.09**
*Speed (20 m sprint)*			
Age	0.207	0.010	**20.09**
Age x SI123 vs. SI4	0.091	0.024	**3.78**
Age x SI12 vs. SI3	0.044	0.025	1.74
Age x SI1 vs. SI2	0.046	0.028	1.65
Gender	0.303	0.006	**50.26**
*PowerLOW (standing long jump)*			
Age	0.213	0.011	**20.23**
Age x SI123 vs. SI4	0.096	0.025	**3.90**
Age x SI12 vs. SI3	0.007	0.026	0.25
Age x SI1 vs. SI2	-0.008	0.029	-0.29
Gender	0.391	0.006	**63.46**
*PowerUP (ball-push test)*			
Age	0.521	0.010	**52.44**
Age x SI123 vs. SI4	0.066	0.023	**2.87**
Age x SI12 vs. SI3	0.065	0.024	**2.64**
Age x SI1 vs. SI2	0.047	0.027	1.73
Gender	0.664	0.006	**113.60**
*Static balance (one-legged-stance)*			
Age	0.148	0.010	**14.28**
Age x SI123 vs. SI4	0.034	0.024	1.40
Age x SI12 vs. SI3	0.013	0.026	0.49
Age x SI1 vs. SI2	0.017	0.028	0.60
Gender	-0.242	0.006	**-39.91**

Note. Age = linear age trend. School social index contrasts: SI123 vs. SI4 = Mean of index categories 1, 2, and 3 vs. index category 4; SI12 vs. SI3 = Mean of index categories 1 and 2 vs. index category 3, SI1 vs. SI2 = Index category 1 vs. index category 2. Bold = |z| > 2

Table 4 shows variance components (VCs) and correlation parameters (CPs) of the LMM. VCs indicated that schools differed in their physical fitness, Covid pandemic effects, and gender effects. CPs were in line with previously reported results [1, 8], indicating that schools with better average physical fitness before the pandemic exhibited more pronounced negative Covid pandemic effects in the respective fitness components (CPs between − 0.53 for powerUP and − 0.44 for speed and powerLOW).

**Table 4:** Child- and school-related variance components and correlation parameters of the linear mixed model

	VC	CP										
								Covid @ crit. date				
		E	C	S	pL	pU	B	E	C	S	pL	pU
**Child**												
E	0.513											
C	0.534	+ 0.57										
S	0.541	+ 0.64	+ 0.68									
pL	0.589	+ 0.61	+ 0.67	+ 0.79								
pU	0.489	+ 0.25	+ 0.47	+ 0.44	+ 0.52							
B	0.549	+ 0.26	+ 0.28	+ 0.27	+ 0.30	+ 0.15						
**School**												
E	0.128											
C	0.166	+ 0.32										
S	0.126	+ 0.30	+ 0.32									
pL	0.067	+ 0.38	+ 0.40	+ 0.40								
pU	0.071	+ 0.18	+ 0.23	+ 0.26	+ 0.23							
B	0.123	-0.02	+ 0.23	+ 0.09	+ 0.13	+ 0.10						
Covid @ crit. date												
E	0.088	**-0.50**	-0.11	-0.13	-0.06	-0.04	+ 0.06					
C	0.172	-0.13	**-0.49**	-0.04	-0.15	-0.15	-0.10	+ 0.12				
S	0.122	-0.14	-0.03	**-0.44**	-0.02	-0.09	-0.02	+ 0.21	+ 0.15			
pL	0.054	-0.19	-0.16	-0.16	**-0.44**	-0.12	-0.02	+ 0.07	+ 0.16	+ 0.21		
pU	0.075	-0.05	-0.05	-0.06	-0.04	**-0.53**	-0.19	+ 0.07	+ 0.18	+ 0.11	+ 0.08	
B	0.151	-0.01	-0.06	-0.04	-0.01	-0.07	**-0.45**	-0.11	+ 0.14	+ 0.02	+ 0.04	+ 0.03
Gender	0.002	-	-	-	-	-	-	-	-	-	-	-

E = cardiorespiratory endurance (i.e., 6-min run), C = coordination (i.e., star-run), S = speed (i.e., 20-m linear sprint), pL = lower limbs muscle power (i.e., powerLOW, standing long jump), pU = upper limbs muscle power (i.e., powerUP, ball-push test), B = static balance (i.e., one-legged-stance test). Covid @ crit. date = Covid pandemic effect estimated at first day of school in school year 2020/21. VC = variance component, CP = correlation parameter. Theoretically relevant correlations are set in bold. LMM random factors: schools (444) and children (108,308), observations = 628,399. VC for Residual = 0.300.

## Discussion

We examined whether (1) physical fitness and (2) pandemic effects differed by schools’ social status, (3) whether negatively affected physical fitness components have recovered after the pandemic, and (4) whether potential rebound effects differed by social status. We used previously analyzed data from cohorts 2016–2022 [8, 26] and added new fitness data from cohort 2023. Positive linear or quadratic secular trends during cohorts 2020–2023 were interpreted as evidence for post-pandemic rebound effects.

As predicted and in line with previous studies [7, 9], children from schools with lower social status tended to exhibit poorer 6-min run, star-run, 20 m sprint, and standing long jump performance relative to children in schools with higher social status. Ball-push test performance did not differ by social status, while one-legged stance test was better in school social status category 3 compared to 1 and 2. Previous research has shown that performances in four weight-bearing EMOTIKON tests (i.e., 6-min run, star-run, 20 m sprint, and standing long jump) were positively correlated, while the ball-push and one-legged stance tests showed lower correlations with the other fitness tests [1, 8, 26]. Higher BMI is positively related to ball-push test performance, but negatively affects performance in running and jumping tests [1, 42, 43, 44]. The poorer performance for low social status schools in running and jumping tests, but not in the other two fitness tests, may therefore be related to the effects of body constitution on test performance [1, 42, 43, 44]. Since ball-push performance reflects muscular power on the one hand, but is also an indicator for overweight and obesity on the other, these two influences may ‘cancel each other out’, which could explain the lack of differences by school social status.

When estimated at the first day of school in school year 2020/21, there were small negative Covid pandemic effects in cardiorespiratory endurance, coordination, speed, and powerUP. These results were similar to those reported in our previous study that included data from cohorts 2016 to 2022 [8]. In the previous study, we also reported better average performance in tests assessing powerLOW and static balance after, compared to before the pandemic onset. However, this pattern of results was likely explained by secular trends independent of the Covid pandemic. In the present report, we adjusted for secular trends by using a regression discontinuity design to estimate pandemic effects at the first day of school in school year 2020/21.

Pandemic effects and cohort trends differed by school social status. First, schools with lower social status exhibited slightly more negative trends in cardiorespiratory endurance and speed before the pandemic. Second, in line with previous research indicating larger pandemic effects in “fitter” schools [1, 8] and in schools with higher socioeconomic background [7], pandemic effects on the star-run and 20 m sprint performance were slightly more pronounced in schools with higher social status. In the other negatively affected fitness components cardiorespiratory endurance and powerUP, there was no evidence for significant differences in pandemic effects across school index categories. However, similar to previous reports [1, 8], results indicated that schools with better average performances exhibited larger negative Covid pandemic effects in all six fitness components.

After an initial decline in performance at the start of the pandemic, coordination and powerUP rebounded, with a slightly larger coordination rebound in schools with high social status. Cardiorespiratory endurance and speed in 2023 remained at levels similar to the pandemic years. Other studies assessing children’s physical fitness development until 2022 reported mixed findings, with evidence for some rebounds after the pandemic [3, 6]. In a large Slovenian study including over 41,000 children and adolescents tested yearly between 2019 and 2022, cardiorespiratory endurance, muscular fitness, speed, neuromuscular and gross motor coordination declined from 2019 to 2020 but rebounded in all physical fitness components. These rebounds, however, were incomplete in most gender-BMI groups, with physical fitness remaining lower in 2022 compared to 2019 [6]. Similarly, Austrian elementary school children still exhibited lower cardiorespiratory endurance, agility and flexibility in 2022 compared to pre-pandemic cohorts [3]. PowerUP, however, was better in 2022 than in pre-pandemic cohorts 2016–2019. Pandemic effects and incomplete rebounds may be associated with pandemic-related losses of sports opportunities. Another potential reason for the incomplete fitness rebounds is the development of post-acute sequelae of SARS-CoV-2 infection (i.e., long Covid), which encompasses a wide range of symptoms like fatigue, respiratory issues (i.e., cough or shortness of breath), headaches, nausea, dizziness, or “brain fog” [45, 46]. Recent meta-analyses reported a prevalence of long Covid symptoms in youth ranging from 23 to 25%, with up to 15% of these children and adolescents experiencing respiratory symptoms [47, 48]. Considering that Covid infections still pose a challenge to population health [49], the missing post-pandemic improvement especially in cardiorespiratory endurance may also be related to long Covid prevalences in child populations.

In the present study, larger pandemic effects and larger subsequent rebounds in schools with higher social status illustrate the importance of a cultural environment and infrastructure providing sports opportunities for children’s fitness. Further, it is possible that some parents in higher social status schools may have been able to provide more support measures after the pandemic, like additional opportunities to be physically active or academic support, that may have contributed to the more positive development of coordination and speed after the pandemic.

Between-school differences in children’s fitness can be related to the socio-structural makeup of the individual schools, but may also reflect region-related differences. For instance, the proximity, accessibility (e.g., public transportation), or variety of sports clubs, and the quality of school sports facilities may differ between regions. In the German State of Brandenburg, communes can be classified either as close to Berlin (“Berliner Umland“) or far from Berlin (“weiterer Metropolenraum”) [50]. The area close to Berlin is strongly integrated with the metropolitan capital city. It exhibits a higher population density [50] and lower average socioeconomic deprivation than the largely rural area far from Berlin [51]. In the school entry examination, regions close to Berlin exhibit a lower percentage of overweight and obese children than regions far from Berlin [52]. Further, previous analyses have shown that third-graders living close to Berlin tend to exhibit better 6-min run, 20m sprint, and standing long jump performance compared to children in regions far from Berlin [53] and that children from rural Brandenburg areas are less often in sports clubs than children in urban areas [54]. Additional analyses (code provided in the OSF repository) therefore tested whether regional fitness differences are explained by schools’ socio-structural composition or indicate additional regional effects on fitness. Indeed, an LMM adjusting for school social status indicated that children in schools close to Berlin exhibited slightly better 6-min run and standing long jump performances than children far from Berlin, which is evidence for additional region effects on fitness independent of school social status effects. Access to affordable sports opportunities and healthy nutrition should be enhanced in socioeconomically disadvantaged schools with lower average fitness, especially in Brandenburg regions far from Berlin. Sports opportunities can include school-based physical activity programs [55, 56], potentially in collaboration with local sports clubs, that might not only positively affect children’s activity and fitness, but also their cognitive performance [57, 58]. Providing healthy and affordable school lunches, creating safe, walkable neighborhoods [59, 60], and informing parents about health benefits of physical activity or the location of local sports opportunities may also enhance children’s activity levels. However, as childhood health disparities are linked to socioeconomic disparities [16, 18, 61], lasting improvements in children’s health likely require addressing underlying socioeconomic inequalities.

As cardiorespiratory endurance and muscular fitness are closely linked to physical health [62, 63] and well-being [64] in youth, children from socioeconomically disadvantaged areas likely are, through no fault of their own, at greater risk of adverse health outcomes. In fact, according to 18,773 voluntary parental reports of children’s body mass and height in EMOTIKON cohorts 2021, 2022 and 2023, around 18% of children in social index category 4 were overweight or obese, compared to 10% of children from school index category 1. As self-reported height and weight were only available from cohort 2021 onwards, we were not able to estimate Covid pandemic effects on children’s body constitution. However, in Berlin, Germany, although physical fitness losses were more pronounced in schools with higher socioeconomic status [7], pandemic-related BMI increases were larger in schools of lower socioeconomic status [14].

Another interesting result of the present study was that the previously reported larger age-related fitness development in the ninth year of life in schools with higher average fitness (i.e., positive correlation between schools’ random intercept and random age effect) [26] was explained by the interaction between age and school social status. In fact, adding age as a random effect for schools did not improve the model fit but led to singularity of a model with the fixed effect school social index. Schools with higher social status exhibited slightly larger developmental rates in the ninth year of life in all fitness components except static balance. This larger age-related development may be related to better access to sports opportunities or higher-quality physical education lessons and school sports [26, 65]. It is also possible that higher overweight and obesity rates in disadvantaged schools “impede” the usually positive effects of age-related growth on children’s fitness, resulting in decreasing age-related fitness development with increasing BMI in third-graders [1].

It is important to note that the school-specific social index we used was based on three different indicators (social welfare [SGB-II] rate, non-German family language rate, and special educational needs rate). While other studies using similar indices have used terms like ‘school social burden’ [9] or ‘socioeconomic background’ [7, 14], we used ‘school social status’ for reasons of brevity and to improve text clarity. Rather than only socioeconomic status, the school social index reflects a broader measure of social disadvantage or social marginalization. Future reports may examine how different indicators of social marginalization may differ in their relationship with children’s physical fitness.

## Conclusions

Negative pandemic effects on coordination and speed were more pronounced in schools with higher social status. There were post-pandemic rebounds in coordination and powerUP, with slightly larger coordination rebounds for schools with higher social status. Differences in Covid pandemic and rebound effects between schools highlight the importance of children’s living environment for their physical fitness development. Enhancing healthy living environments by increasing access to affordable sports opportunities and to affordable healthy food options in disadvantaged areas may help compensate physical fitness differences. Although pandemic effects were small, absence of evidence for rebounds in cardiorespiratory endurance and speed as of 2023 may indicate long-term consequences of pandemic-related movement restrictions. Regular assessments of children’s physical fitness are needed to examine how physical fitness develops throughout the next years.

## Data Availability

Supplementary material, data, as well as R and Julia scripts are available in the Open Science Framework (OSF) repository: https://osf.io/5273n/.

## List of abbreviations

CP: Correlation parameter
LMM: Linear mixed model
RDD: Regression discontinuity design
VC: Variance component

## Contrasts for four-level factor school social status

SI123 vs. SI4: Mean of categories 1, 2, and 3 vs. category 4
SI12 vs. SI3: Mean of categories 1 and 2 vs. category 3
SI1 vs. SI2: Category 1 vs. category 2
